# Optimal Codon Identities in Bacteria: Implications from the Conflicting Results of Two Different Methods

**DOI:** 10.1371/journal.pone.0022714

**Published:** 2011-07-28

**Authors:** Bin Wang, Zhu-Qing Shao, Ying Xu, Jing Liu, Yuan Liu, Yue-Yu Hang, Jian-Qun Chen

**Affiliations:** 1 State Key Laboratory of Pharmaceutical Biotechnology, School of Life Sciences, Nanjing University, Nanjing, Jiangsu Province, China; 2 Institute of Botany, Jiangsu Province and Chinese Academy of Science, Nanjing, Jiangsu Province, China; Columbia University, United States of America

## Abstract

A correlation method was recently adopted to identify selection-favored ‘optimal’ codons from 675 bacterial genomes. Surprisingly, the identities of these optimal codons were found to track the bacterial GC content, leading to a conclusion that selection would generally shape the codon usages to the same direction as the overall mutation does. Raising several concerns, here we report a thorough comparative study on 203 well-selected bacterial species, which strongly suggest that the previous conclusion is likely an illusion. Firstly, the previous study did not preclude species that are suffering weak or no selection pressures on their codon usages. For these species, as showed in this study, the optimal codon identities are prone to be incorrect and follow GC content. Secondly, the previous study only adopted the correlation method, without considering another method to test the reliability of inferred optimal codons. Actually by definition, optimal codons can also be identified by simply comparing codon usages between high- and low-expression genes. After using both methods to identify optimal codons for the selected species, we obtained highly conflicting results, suggesting at least one method is misleading. Further we found a critical problem of correlation method at the step of calculating gene bias level. Due to a failure of accurately defining the background mutation, the problem would result in wrong optimal codon identities. In other words, partial mutational effects on codon choices were mistakenly regarded as selective influences, leading to incorrect and biased optimal codon identities. Finally, considering the translational dynamics, optimal codons identified by comparison method can be well-explained by tRNA compositions, whereas optimal codons identified by correlation method can not be. For all above reasons, we conclude that real optimal codons actually do not track the genomic GC content, and correlation method is misleading in identifying optimal codons and better be avoided.

## Introduction

It has long been realized that mutation and selection are two major forces affecting codon usage biases [1]–[4], and an organism's codon usage pattern is determined by the combined influences of both forces on synonymous codon choices. The ‘optimal’ codons, designated by Ikemura [3] as preferred codons in high expression genes, would signal the influences of translational selection on codon choices [5]–[7]. When selection is weak or even absent, codon usages in an organism would be mainly determined by complex mutational forces together with drift [8], [9].

Many studies have focused on prokaryotic genomes to investigate how mutation and selection would influence the codon usages within or among species [6], [7], [10]–[14]. It was found that variant codon usage patterns among different bacterial species are predominantly determined by genome-wide mutational processes, such as overall nucleotide substitution bias, context-dependent mutational bias, and repair-associated bias [10], [11], [15]. After the mutational forces coarsely set the genome-wide codon usage pattern for a given species, selection would further increase the ‘optimal’ codon frequencies in relevant genes, mainly in the high expression ones [11]. The strength of selection force, however, is highly variable among species. A best example illustrating this came from a study by Sharp and his colleagues [6]. Among the 80 bacterial species examined, 30% genomes showed no significant evidence of selection in effective. While among the rest of genomes studied, the estimated strength of selection is highly positively correlated with the total tRNA gene numbers. Rocha [12] had also investigated 102 bacterial species and divided them into fast-growers and slow-growers according to their minimal generation times. It was found that fast-growers overall have more abundant (with a median of 61) tRNA genes than slow-growers (with a median of 44); also fast-growers suffer stronger selective influences on their codon usages in high expression genes [12].

By definition, if a bacterial species showed significantly different codon usages between high expression genes and low expression ones, it would signal the presence of selection. Based on this, one straightforward method of identifying ‘optimal’ codons was defined. As described by several studies [6], [13], [14], this method assumes that codon usages in high expression genes, such as ribosomal protein genes and elongation factor genes, are influenced by both mutation and selection forces; while the codon usages in other genes as a whole can be reasonably thought to be influenced by mutation force only. By comparing the relative codon frequencies of two sets of genes, one is then able to identify selection-favored ‘optimal’ codons in different amino acid families. To be convenient, we call this simple way of identifying optimal codons as “comparison method” hereafter.

In addition, there is another way to identify optimal codons in literature. It is mainly built on a basic idea that has been documented in several early researches [16]–[18]: an ‘optimal’ codon would increase its frequency when genes become more biased in the choice of favored codons overall. With the popularization of convenient ways to measure a gene's bias level, such as *Nc* or *Nc′* (effective number of codons, [19], [20]), ‘optimal’ codons were identified (through correlation tests) as those showing a statistically significant increase in frequency between lowly- and highly-biased genes. Here we call this method as “correlation method”. A large scale application of this method came from a very recent study [21]. By conducting correlation tests, the authors identified selection-favored ‘optimal’ codons from as many as 675 bacterial genomes. Surprisingly, the identities of detected optimal codons were found to track the bacterial GC content, leading to a conclusion that selection force would act generally in the same direction as the overall mutational bias [21].

Such conclusion, however, does not look well-founded in our view due to several concerns. Firstly, the study did not preclude species that are suffering weak or no selective influences on their codon usages. Previously, Sharp et al. [6] had identified 24 species in which selection has no significant effects on their codon usages. Also, Rocha [12] documented 41 slow-growing bacterial species that likely suffer weak selection on their codon usages. Nearly all these species, as well as their close relatives, were included in Hershberg and Petrov's study [21]. Optimal codons identified from these species should be treated in caution, since they are more likely representing effects of other forces (variation caused by complex mutational forces or drift) rather than selection. Secondly, Hershberg and Petrov [21] only used correlation method to identify optimal codons, without considering the more straightforward comparison method to test the reliability of inferred optimal codons. In fact, as far as we know, it has never been compared in detail for the effects of “comparison method” and “correlation method” in identifying optimal codons. Would both methods lead to same optimal codon identities? If not, which method would be more reliable and efficient? Finally, although the authors claimed that selection would shape the codon usages to the same direction as overall mutation does in prokaryotes, they did not offer any reasonable explanation on the very nature of such selection.

To better handle these concerns, we carefully selected two sets of data: one includes 102 bacterial species, all of which have high tRNA gene numbers (HTN) in their genomes; the other includes 101 bacterial species, all of which own low tRNA gene numbers (LTN). Since the total tRNA gene number is positively correlated with the selection strength and a median of 61 and 44 was respectively identified for fast-growers and slow-growers [6], [12], we expect that the HTN group (>65 total tRNA genes in every species) would overall suffer strong selective influences on their codon usages, while the LTN group (<45 total tRNA genes in every species) would more likely suffer weak selective influences. Both comparison method and correlation method were adopted to identify optimal codons for these two sets of data. The obtained results were then analyzed in detail to compare their differences between groups and between methods.

## Results and Discussion

### Optimal codons identified by two methods are highly conflicting

**Figure 1:**
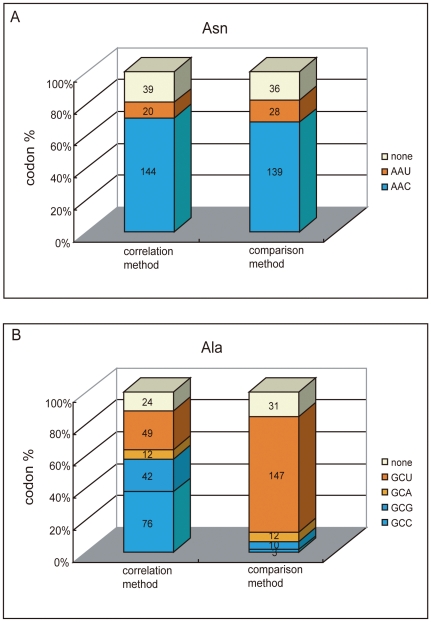
Overall result of optimal codon identities via two methods. All 203 selected species were included. Different colors were used to label different codon identities, as well as the case in which no optimal codon can be identified. The total numbers of species sharing a same codon identity were also labeled. A. The data obtained from Asparagine family was shown. B. The data obtained from Alanine family was shown.

A total of 203 species (Table S1) were analyzed in this study. For each species, its optimal codons via both correlation method and comparison method were identified (Table S2). Here we take Asn family and Ala family as two examples (one representing the two-fold degenerate families and the other representing the four-fold degenerate families) to illustrate the overall results of two methods in identifying optimal codons. In Asn family (Figure 1A), both correlation method and comparison method predominantly identified AAC as the optimal codon (in 144 and 139 out of 203 species, respectively). The overall consistency on optimal codon identities between two methods is 76.8% for this family. In Ala family (Figure 1B), however, the inconsistency is apparent. Correlation method identified GCC, GCG, GCA, and GCU as optimal codon in 76, 42, 12 and 49 species, respectively; while comparison method predominantly identified GCU as optimal codon in 147 species. The consistency level between two methods in this family is only 24.6%. In other families, conflicting results on optimal codon identities between two methods were also obtained, and overall the consistency level is higher in two-fold degenerate families than in four-fold degenerate families (Table 1).

**Table 1:** Percentages of consistency between optimal codons identified by two methods.

Amino acid families	All species (203)	HTN group (102)
Asn	76.8%	93.1%
Asp	61.6%	66.7%
Cys	52.7%	47.1%
His	74.4%	80.4%
Phe	67.0%	85.3%
Tyr	74.9%	85.3%
Gln	54.7%	43.1%
Glu	63.1%	65.7%
Lys	51.2%	46.1%
Ile	58.6%	62.7%
Ala	24.6%	12.7%
Gly	36.0%	34.3%
Pro	37.4%	38.2%
Thr	21.7%	12.7%
Val	26.6%	19.6%
Arg	37.9%	49.0%
Leu	38.9%	52.0%
Ser	28.1%	26.5%

HTN group includes 102 bacterial species with high tRNA numbers (>65) in their genomes.

One possibility causing such conflicting results is that the 203 studied species include both HTN and LTN species. HTN species are expected to suffer strong selection influences on their codon usages, while LTN species are not. Thus optimal codons identified from some LTN species may not represent selective influences at all. In such cases, different methods might reach conflicting results. To preclude such possibility, we kept the 102 HTN species only and calculated the consistency level again. It was found that in two-fold degenerate families, such as Asn, Asp, His, Phe, and Tyr, the consistency level indeed increased, but in many other families, especially in the four-fold degenerate families, such as Ala, Thr, and Val, the consistency level was even reduced (as low as 12.7%, Table 1). Such conflicting results strongly suggest that correlation method and comparison method are actually incompatible in identifying optimal codons, even when only species under strong selective influences are considered (HTN group, 102 species).

### Does optimal codon identity really follow bacterial GC content?

**Figure 2:**
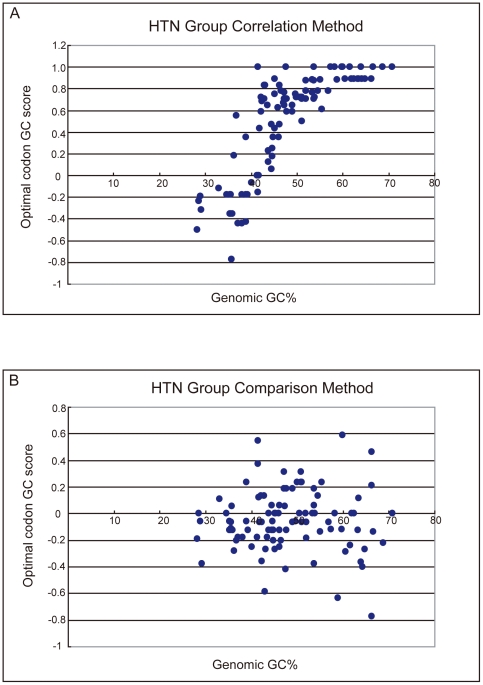
Correlation between GC scores of optimal codons and genomic GC content. The most GC-rich codons in each codon family received a score of 1, the most AT-rich codons in each family received a score of −1. Arginine and Leucine codons of intermediate GC content received a score of 0. For each genome, the average GC score for all the identified optimal codons were calculated. The scores were then plotted against the genomic GC content. Only 102 species with high tRNA gene numbers (HTN) are included. A. Correlation method was adopted. B. Comparison method was adopted.

The most surprising pattern observed by the previous study [21] is that the optimal codon identities via correlation method generally track the genomic GC content in bacteria. In last section, we have shown that the optimal codons identified by two methods are largely different; now we will show that in general, the optimal codon identities via comparison method would not follow bacterial GC content at all. Following the description of previous study [21], we calculated the optimal codon GC scores for all the HTN species and plotted the scores against the genomic GC contents. As Figure 2A showed, for correlation method, the optimal codon GC score does seem to follow the bacterial GC content in general (*r_spearman_* = 0.855, p<0.001), as previous study has reported. However, Figure 2B showed that when comparison method is adopted, the optimal codon GC score has no correlation with genomic GC content at all (*r_spearman_* = 0.08, p = 0.42).

In the previous study, the authors also checked all individual families and found that when GC content is low, U- or A-ending codons are likely identified as optimal and with GC content increasing, G- or C-ending codons are more prone to be optimal. However, below we will demonstrate that when only species of HTN group are considered, the trend is often not true in individual families. Moreover, the biased pattern observed by previous study is likely contributed by species of LTN group, which generally suffer weak or no selective influences.

#### Optimal codon identities in HTN group

**Figure 3:**
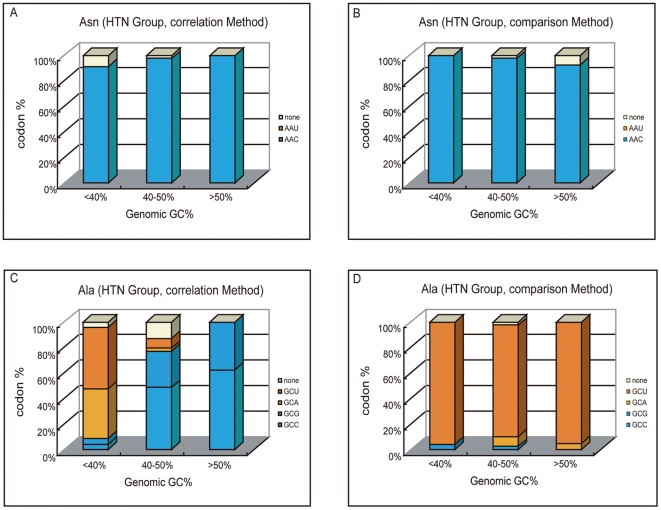
Optimal codon identities under different genomic GC levels. The 102 HTN species were divided into three groups according to their genomic GC contents (23 species, <40%; 39 species, 40–50%; 40 species, >50%). In each group, the different optimal codon identities were shown in different colors. A. The optimal codons identified by correlation method for Asparagine family were shown. B. The optimal codons identified by comparison method for Asparagine family were shown. C. The optimal codons identified by correlation method for Alanine family were shown. D. The optimal codons identified by comparison method for Alanine family were shown.

Figure 3A represents the case of Asn family when correlation method is applied. Among a total of 102 species in the HTN group, AAC was identified as optimal codon in 99 species, including 21 species with GC content lower than 40%. If optimal codon identity does follow GC content as reported previously, one would expect to see AAU as optimal codon in those species with genomic GC content lower than 40%. No such case was detected for Asn family. Similarly, within Asp, His, Phe, and Tyr families, U-ending codons were never identified as optimal in a total of 23 HTN species with GC content lower than 40% (Figure S1). Thus, these five U+C codon families serve as exceptions to the pattern previously reported, because their optimal codon identities do not change with the genomic GC content in the HTN group. However, in other families, optimal codons identified by correlation method indeed follow genomic GC content. Figure 3C represents such an example (Ala family). When genomic GC content is less than 40%, identified optimal codons are mainly GCA and GCU. With GC content further increases, GCC and GCG codons are largely identified as optimal. Similar cases can also be found in Gln, Lys, Gly, Pro, Thr, and Val families (Figure S1), and the increasing pattern observed in Figure 2A should be largely contributed by these families.

On the other hand, Figure 3B and 3D represent the cases of Asn and Ala families when comparison method was used. At three different GC levels, AAC was always identified as the optimal codon for Asn family, and GCU, occasionally GCA, was identified as optimal codon for Ala family. Obviously in both cases, the optimal codon identities do not track the genomic GC content. This conclusion can also be safely extended to many other families, such as Asp, Cys, His, Phe, Tyr, Gly, Thr, and Val (Figure S1). The pattern observed in Figure 2B reflects a combined effect of all these families.

#### Optimal codon identities in LTN group

**Figure 4:**
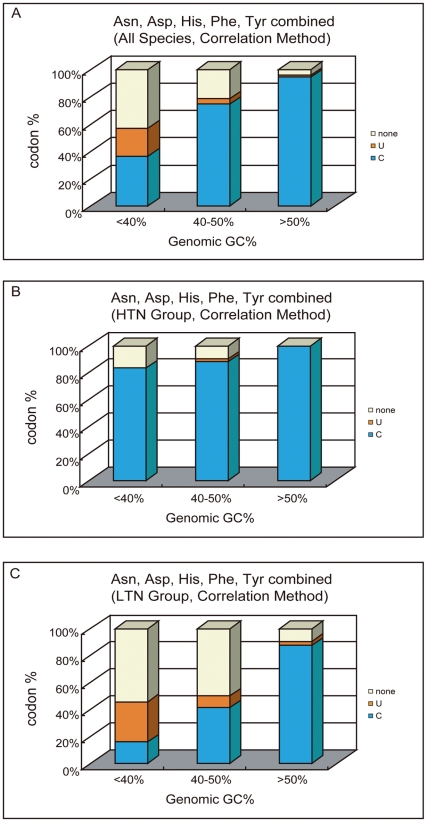
The observed pattern between optimal codon identities and GC content may depend on data selection. Five C+U codon families (Asparagine, Aspartic acid, Histidine, Phenylalanine, and Tyrosine) were combined. A. When all 203 selected species are included, the optimal codon identities seem track the genomic GC content. B. When only the 102 HTN species are included, the optimal codon identities do not track the genomic GC content since U-ending codons were never identified as optimal among species with <40% genomic GC content. C. When only the 101 LTN species are included, the optimal codon identities follow the genomic GC content.

Although the selective influences on codon usages are expected to be weak in LTN group, both correlation method and comparison method are still able to identify ‘optimal’ codons for many species (Table S2). It is possible that in some cases, the selective influences on codon usages are still significant enough for proper identification of optimal codons, but more likely in many other species, the codon usage variations are mainly caused by forces other than selection. In such cases, the results would be inaccurate and not meaningful. In our opinion, it is better not include any species of this group when drawing a conclusion on optimal codon identities. The previous study [21] clearly did not filter these species out. Concerned by this, here we tested the influences of including LTN species on the general pattern of optimal codon identities. Earlier we have shown that when only HTN group is considered, C-ending codons were consistently identified as optimal in Asn, Asp, His, Phe and Tyr families. Combining the data of these five families together, we can reach a general conclusion that optimal codon identities in these five families actually do not follow the genomic GC content when correlation method is adopted (Figure 4B). However, when the data of LTN group is incorporated (Figure 4A) or just considered alone (Figure 4C), a contrary conclusion that optimal codon identities would follow the genomic GC content in these families can be drawn. It is mainly because the U-ending codons in these families are often identified as optimal for LTN species, especially when their genomic GC contents are lower than 40%. Such pattern in LTN species can also be found in many other families (Figure S2). Taken together, these results suggest to us that the major pattern observed in previous study [21] is at least partially contributed by unreliable results obtained from species under weak or no selections.

### Optimal codon identities and tRNA compositions

As demonstrated earlier, the optimal codons identified by correlation method and comparison method are highly conflicting (Figure 1, Table 1). Even when only species of HTN group were considered, the conflicting results are still apparent, especially in four-fold degenerate families (Table 1). These facts strongly suggest to us that between correlation method and comparison method, at least one is misleading in identifying real optimal codons. Next, we will use examples to demonstrate that from tRNA's point of view, the optimal codons identified by comparison method can be well-explained, but the optimal codons identified by correlation method often can not.

First we look at the Asn family. For 102 HTN species, both correlation method and comparison method identified only AAC as the optimal codon for this family (in 99 and 98 out of 102 species, respectively). This highly consistent result can be explained from the tRNA's point of view. In genomes of all selected species, there is only one type of tRNA gene available for Asn, tRNA^Asn^(GUU) (see Table S1). Previous studies have revealed that in such case, codon AAC should be selectively favored, because during the translation the anticodon (GUU) of the tRNA would pair more rapidly with codon AAC rather than with codon AAU [6], [13], [22]. Therefore, when selective influences on codon usages are expected to be strong, both correlation method and comparison method seemed perform fine in identifying correct optimal codon in this two-fold degenerate family.

However, in four-fold degenerate families, e.g. Ala family, the correlation method identified various optimal codons and in general the identity follow the bacterial GC content (Figure 3C); while the comparison method mainly identified GCU as optimal codon (Figure 3D). By checking the tRNA compositions of all 102 HTN species (see Table S1), we found that two types of tRNA genes are mainly present for this family: tRNA^Ala^(UGC) (present in all 102 species) and tRNA^Ala^(GGC) (present in 81 species). In such cases, as reported by Ran and Higgs [14], the codon GCU would be the most likely favored codon because both anticodons can pair with it in high efficiency. This is consistent to the results of comparison method, which predominantly identified GCU as the optimal codon. Differently, correlation method identified codon GCG as optimal in 27 species and codon GCC as optimal in 45 species. Carefully checking these species found that in most cases, the dominant tRNA species for this family is tRNA^Ala^(UGC), which would pair with GCC or GCG in low efficiency [14]. Thus, from the tRNA's point of view, these species seem unlikely to have GCC or GCG as their optimal codons. In other four-fold degenerate families, including Gly, Pro, Thr, and Val families, similar analyses were also conducted and the conclusions are generally the same: correlation method identified C- or G-ending codons as optimal in much more cases than it should according to their tRNA compositions.

### An internal problem of correlation method leads to biased optimal codon identities

Above findings impelled us to carefully review the process of correlation method in identifying optimal codons. In this method, the ‘optimal’ codons are mainly identified by conducting correlation tests between synonymous codon frequencies and estimated gene bias levels. The highly-biased genes are assumed to use more optimal codons overall, and the key step of this method is to accurately measure the gene bias level under selective influences only. Next, we will show that the problem is right at this step.

**Figure 5:**
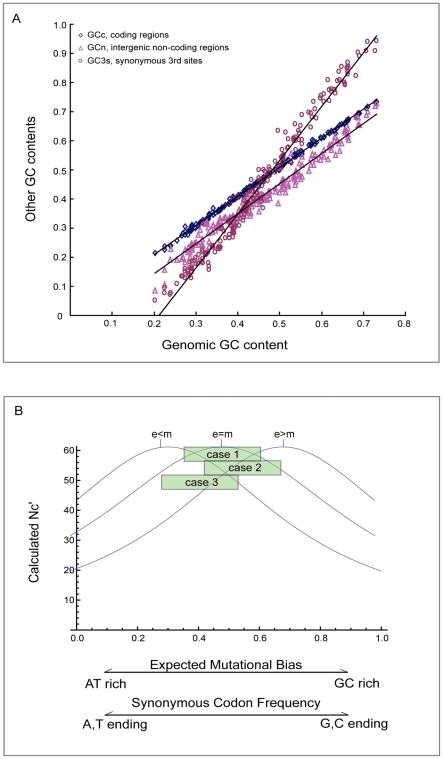
An internal problem of correlation method can lead to biased optimal codon identities. A. As genomic GC content increases, the GC contents of coding sequences (GCc), non-coding sequences (GCn), and synonymous 3^rd^ codon position (GC3s) would change in different paces. B. Calculated *Nc′* values change with relative synonymous codon frequency. The three curves represent three conditions: expected background mutational bias smaller than, equal to, or larger than real mutational bias. In case 1, selection has no effect on codon usage. In case 2, selection favors G, C-ending codons. In case 3, selection favors A, T-ending codons.

To reach the goal of measuring a gene's bias level driven by selection force only, the previous study [21] adopted *Nc′*, a way to estimate gene bias level by precluding the background mutational influences [20]. However, to calculate *Nc′*, one has to define the background mutational bias first. Due to the complex nature of mutational dynamics, e.g. content-dependent mutation, it is extremely difficult to estimate the mutational bias in an accurate way. Therefore the adopted estimation criteria may inevitably deviate from the real mutational bias. In the previous study [21], the authors followed the default setting of ENCprime software [20] to calculate *Nc′*, so the background mutational bias was estimated from the nucleotide frequencies of each coding sequences. Figure 5A shows how GC contents of coding sequences (GCc), non-coding sequences (GCn), and synonymous 3^rd^ sites (GC3s) would change with the genomic GC content (GCg). The relationship between GCc (approximately the standard adopted by previous study to estimate the background mutational influences) and GC3s (approximately reflecting the combined effects of real mutational influences on synonymous 3^rd^ codon positions of all genes plus selective influences on synonymous 3^rd^ codon positions of a subset of high expression genes) is critical. Although the real level of mutational bias (GCm) acting on codons is unknown, it is likely smaller than GCc at low genomic GC level and larger than GCc at high genomic GC level, as indicated by the GC3s line in Figure 5A. Therefore we speculate that in the previous study, when calculating the *Nc′* to measure the gene bias level, the background mutational influences were overestimated for GC-poor bacteria and underestimated for GC-rich bacteria, and the resulted *Nc′* values were inaccurate.

We further plotted the curves of *Nc′* value against synonymous codon frequency under three conditions: when an expected GC mutational bias (GCe) is smaller, equal to, or larger than the real mutational bias (GCm). As presented in Figure 5B, the middle curve represents a nice accurate estimation of background mutational bias (GCe = GCm). Let's take the Asn family as an example and suppose there are three possible cases. In case 1, selection has no effect on codon usage; in case 2, selection favors C-ending codons and is likely to increase the frequency of AAC codon in many genes; and in case 3, selection favors AAU instead. By examining the correlations of calculated *Nc′* values and codon frequencies, one can clearly detect negative correlations for AAC codon in case 2 and for AAU codon in case 3. Therefore for middle curve, everything is fine and the identified optimal codons really represent the direction of the selection force. What if the mutational bias is underestimated (GCe<GCm), such as in GC rich bacteria? The left curve represents such a condition. The calculated *Nc′* values would deviate from the original values due to the deviation of estimated mutational bias from the real one. In all three cases, the frequency ranges of AAC codon are now located on the right half of the curve and one can probably detect negative correlations for AAC codons for all cases. However, AAC should not be identified as selection-favored codons in case 1 and case 3. Simply to say, because the background mutation is underestimated, part of the mutational effect on codon usage was not corrected out and counted as the effect of selection, leading to more C- or G-ending codons identified as optimal. Similarly, if the mutational bias is overestimated (GCe>GCm, right curve), AAU is likely to be identified as the optimal codon and this is wrong for case 1 and case 2. In reality, when the genomic GC content is at a moderate level, the estimated criteria may not deviate too much from the real mutational bias; but the chance of identifying incorrect optimal codons quickly becomes higher when the genomic GC content increases (more G-, C-ending codons would be mistakenly identified as optimal) or decreases (more A-, U-ending codons would be mistakenly identified as optimal). This explained why the previous study observed the surprising pattern: the optimal codon identity tracks the bacterial GC content. However, as reasoned above, the pattern is more likely an illusion caused by the internal problems of correlation method at the step of calculating gene bias levels.

The phenomenon of codon usage bias has been surveyed in a wide range of organisms. As two major forces affecting codon usages, mutation and selection have all along been investigated for their separate effects on codon choices. A recent study, however, revealed an unexpected connection between effects of these two forces: selection would favor codons which track genomic GC content. To verify and explore such a finding, in this study, we carefully selected two sets of bacterial species and adopted two different methods to identify their optimal codons. Based on the obtained results, several important implications were revealed: Firstly, for species under weak or no selective influences on their codon usages, it makes little sense to use either correlation method or comparison method to identify their optimal codons because the results are unreliable and likely to be incorrect. Secondly, correlation method and comparison method identified highly conflicting optimal codons, and from the tRNA's point of view, the results of correlation method seem strange and unexplainable. Third, correlation method was found to contain a severe internal problem at the step of calculating gene bias level, which would lead to biased optimal codon identities that are tracking genomic GC contents. Therefore, the correlation method should be adopted in caution in the future to identify optimal codons.

## Materials and Methods

### Data Used

The completed genome sequences of the 203 selected bacterial species were downloaded from the NCBI FTP server. (ftp://ncbi.nlm.nih.gov). Their detailed information, including names, groups, GC contents, and most importantly, tRNA compositions, were given in Table S1. The data is consists of two groups: The HTN group includes 102 species, all having more than 65 total tRNA genes in their genomes. The LTN group includes 101 species, all having lower than 45 total tRNA genes in their genomes.

### Identification of optimal codons using correlation method

The basic idea and logic of the correlation method is that an optimal codon would increase its frequency when genes become more biased in the choice of favored codons overall. For each of the selected genomes (Table S1), we first extracted all the protein coding genes to measure their bias levels. As in the previous study [21], *Nc′* [20] was used and it would measure the gene bias level after precluding a defined background mutational influences. Following the default settings of the ENCprime package [20], the *Nc′* values were calculated, but genes shorter than 600 bp were excluded because *Nc′* calculation for short genes is usually inaccurate. For each genome, we also calculated the relative frequencies of each codon in its family for all selected coding genes. The spearman correlations between the frequencies of each codon and gene bias level (*Nc′*) were then examined using the R statistical package. The optimal codon for each codon family was then identified as the one showing the strongest and most significant negative correlation with the *Nc′* (for details, see [21]). All obtained optimal codons from all families for all species were then listed in Table S2 for further analyses.

### Identification of optimal codons using comparison method

By its original definition, optimal codons can be identified by simply comparing the codon usage frequencies between high and low expression genes [3], because presumably, the frequencies in the low expression genes are determined mainly by mutation, whereas the frequencies in the high expression genes are determined by both mutation and selection. Therefore, an optimal codon that is under positive selection would have higher usage frequency in the high expression genes. Following the previous studies [6], [13], [14], a total number of 40 genes, including 3 elongation factor genes and 37 ribosomal protein genes, are regarded as high expression genes in a genome and all the remaining genes can be reasonable thought as low expression ones. For each family, the frequencies of all its synonymous codons were then calculated in both groups of genes and the optimal codon was identified as the one showing the significant increases in frequency from low to high expression genes (Chi-square test). When there are two or more codons showing significant frequency increases, the optimal codon was identified as the one for which the frequency ratio in high to low expression genes is the highest. The final identified optimal codons for all species were also listed in Table S2.

### Analyzing the identified optimal codons by two methods

The overall optimal codon identities via both methods were compared for each amino acid family and their consistency level were calculated for all 203 selected species and also for the 102 HTN species separately. To check whether in HTN group, the identified optimal codons via two methods would overall track the genomic GC content, as previous study did [21], we gave a score of 1 to the identified GC-rich codons, a score of −1 to AT-rich codons, and a score of 0 to the intermediate codons, which only exist in Leucine and Arginine. We then calculated the average GC scores for all identified optimal codons in each species, plotted the scores against the genomic GC contents, and conducted the spearman correlation tests. Further, we checked the relationship between optimal codon identities and GC contents in individual families. For both methods and both groups (HTN and LTN), the optimal codon identities for species with three different GC content levels (smaller than 40%, between 40–50%, and larger than 50%) were investigated.

### Plotting *Nc′* against synonymous codon frequency

To understand why correlation method would result in highly biased optimal codon identities, we plotted the *Nc′* against synonymous codon frequency under three different conditions: estimated background mutational bias (GCe) is smaller than, equal to, or larger than the real mutational bias (GCm). The *Nc′* calculation formulas in the original study [20] were followed and the plots were drawn by using Wolfram Mathematica 7. It should be noted that the x axis of the plot has two meanings: either the synonymous codon frequency or the expected GC level for mutational bias.

## Supporting Information

Table S1 **The 203 bacterial genomes analyzed.**
(XLS)

Table S2 **Optimal codons of 18 degenerate families identified by correlation method and comparison method.**
(XLS)

Figure S1 **Optimal codon identities of HTN group (both correlation and comparison methods) at different genomic GC levels.** Two-fold and four-fold degenerate families (except Asn and Ala) were shown.
(XLS)

Figure S2 **Optimal codon identities of LTN group via correlation methods track the genomic GC content.** Two-fold and four-fold degenerate families were shown.
(XLS)
